# Supplementary material for: Fast and exact stochastic simulations of epidemics on static and temporal networks
Source: PLoS Comput Biol. 2025 Sep 15;21(9):e1013490. doi: 10.1371/journal.pcbi.1013490 (PMC12448994; doi:10.1371/journal.pcbi.1013490)
Supplement: S1 Algorithms — (PDF) [file pcbi.1013490.s001.pdf]

Fast and exact stochastic simulations of epidemics on static and temporal networks
Supplemental Information I: Algorithms

Samuel Cure[1], Florian G. Pflug[1], Simone Pigolotti[1*]

1 Biological Complexity Unit, Okinawa Institute of Science and Technology, Onna, Okinawa 904-0495, Japan.

* simone.pigolotti@oist.jp

We here describe the NEXT-Net algorithm for static and temporal networks in greater detail and provide pseudo-code. This document is organized as follows. In Section 1, we briefly introduce the representation of networks for the purpose of our algorithms. In Section 2, we explain the representation of transmission and recovery time distributions. We then present the next-reaction based NEXT-Net algorithm for static networks in Section 3 and discuss its computational complexity. Finally, we present the temporal NEXT-Net algorithm in Section 4.

1 Networks

In the simulation algorithms discussed below, networks are accessed through an abstract interface which offers the following procedures: NetworkSize returns the number of nodes in the network, NodeDegree(n) returns the out-degree of node n, and Neighbor(n, l) returns a tuple (m, w) comprising the l-th neighbor m of node n and the weight w of link (n, m). This interface treats networks as directed graphs, meaning that it distinguishes the link (i, j) connecting source i to target j from the link (j, i) connecting source j to target i. In our C++ implementation, this abstract interface is realized as an abstract base class. Specific types of networks such as Erdős–Rényi, Barabási–Albert, lattices and empirical networks defined by an adjacency list are implemented as separate classes, and thanks to this abstract interface can be used with all of the algorithms presented hereafter.

2 Probability distributions

The NEXT-Net algorithm allows arbitrary probability distributions of a variable $\tau \in [0, \infty]$ to be used as transmission time distribution $\psi(\tau)$ and recovery time distribution $\rho(\tau)$. Infinity is explicitly included in the domains of these distributions, and represents the case of no transmission along a particular link and no recovery of a particular node, respectively. We write $\Psi(\tau)$ for the survival function associated with the density $\psi(\tau)$ assuming $w_{ij} = 1$. Equation (1) implies that

$$\Psi(\tau) = \exp\left(-\int_0^\tau \lambda(\tau')d\tau'\right), \qquad \psi(\tau) = -\Psi'(\tau) = \lambda(\tau)\Psi(\tau) \tag{1}$$

where $\lambda(\tau)$ is the hazard rate function which describes the instantaneous rate of transmission at time τ after infection. Any distribution can be expressed in the form of Eq. (1) by setting $\lambda = \psi/\Psi$. The probability of eventual transmission along a specific

link is $p_\psi = \int_0^\infty \psi(\tau) = 1 - \Psi(\infty)$. This probability is less than one (and $\psi(\tau)$ therefore not normalized) if $\int_0^\infty d\tau \lambda(\tau) < \infty$.

To simulate epidemics on weighted and temporal networks, our algorithms requires samples from modified transmission time distributions with a shifted and scaled hazard rate function. We have introduced in the Main Text the density $\psi(\tau \,|\, w)$ for a link such that $w_{ij} = w$. Here we extend this two a two-parameter distribution $\psi(\,\cdot\,|\, w, t)$ defined by shifting $\lambda(\tau)$ by t and scaling it by w. The distribution has survival function and density

$$\Psi(\tau \,|\, w, t) = \exp\left(-m \int_0^\tau \lambda(t + \tau')d\tau'\right) = \left(\frac{\Psi(t+\tau)}{\Psi(t)}\right)^m, \tag{2}$$
$$\psi(\tau \,|\, w, t) = m\lambda(t+\tau)\Psi(\tau \,|\, w, t).$$

Transmission and recovery time distributions are typically continuous, i.e. possess a continuous density. NEXT-Net also supports discrete distributions; in that case we define $\Psi(\tau)$ to be the probability that the transmission is greater or equal than τ.

NEXT-Net accesses this two-parameter family of distributions over some base distribution ψ through an abstract interface that offers procedures $\textsc{DrawTime}_\psi(w, t)$ to sample from $\psi(\,\cdot\,|\, w, t)$ and $\textsc{HazardRate}_\psi(\tau)$ to evaluate $\lambda(\tau) = \psi(\tau)/\Psi(\tau)$. Some of the other algorithms implemented in the NEXT-Net C++ Library require some additional procedures like $\textsc{Density}_\psi(\tau, w, t)$ to evaluate $\psi(\tau \,|\, w, t)$, $\textsc{Survival}_\psi(\tau, w, t)$ to evaluate $\Psi(\tau \,|\, w, t)$ and $\textsc{Quantile}_\psi(p, w, t)$ to evaluate the inverse $\Psi^{-1}(\tau \,|\, w, t)$. The REGIR algorithm also requires $\textsc{HazardBound}(\tau)$ to evaluate $\max_{\tau' \in [0,\tau]} \lambda(\tau')$. Different base distributions are implemented as separate classes which implement these functions. Currently NEXT-Net provides the following distributions

Exponential Exponentially-distributed transmission time with non-normalized density $\psi(\tau) = p_\psi \lambda e^{-\lambda\tau}$, parametrized by rate λ and probability $p_\infty = 1 - p_\psi$ of no infection. Corresponds to constant infectiousness (hazard rate) $\lambda(\tau) = \lambda$ in the case of $p_\infty = 0$.

Weibull Weibull-distributed transmission time with non-normalized density $\psi(\tau) = p_\psi \alpha \theta^{-\alpha} \tau^{\alpha-1} e^{-(\tau/\theta)^\alpha}$, parametrized by shape α, scale θ, and probability $p_\infty = 1 - p_\psi$ of no infection. Corresponds to $\lambda(\tau) = p_\psi \alpha \theta^{-\alpha} \tau^{\alpha-1}$, i.e. an infectiousness (hazard rate) which grows/declines with exponent $\alpha - 1$ in the case of $p_\infty = 0$.

Gamma Gamma-distributed transmission time with density non-normalized $\psi(\tau) = p_\psi \theta^{-\alpha} \tau^{\alpha-1} e^{-\tau/\theta}/\Gamma(\alpha)$ where shape $\alpha = \mu^2/\sigma^2$ and scale $\theta = \sigma^2/\mu$ so that the distribution is parametrized by its mean μ, variance σ^2 and probability $p_\infty = 1 - p_\psi$ of no infection.

Lognormal Log-normally distributed transmission time with non-normalized density $\psi(\tau) = e^{-(\ln\tau - m)^2(2s^2)}/\big(\tau\sigma\sqrt{2\pi}\big)$ where log-mean $m = 2\log(\mu) - \log(\mu^2 + \sigma^2)/2$ and log-variance $s^2 = \log(1 + \sigma^2/\mu^2)$ so that the distribution is parametrized by its mean μ, variance σ^2 and probability $p_\infty = 1 - p_\psi$ of no infection.

Empirical infectiousness Transmission time with non-normalized density $\psi(\tau) = \lambda(\tau)\exp\big(-\int_0^\tau \lambda(\tau')d\tau'\big)$ where the infectiousness (hazard rate) is specified by vectors (τ_i), (λ_i) with $\tau_1 \leq \cdots \tau_n$ and $\lambda_i = \lambda(\tau_i)$. Between the specified points, $\lambda(\tau)$ is interpolated linearly, for $\tau > \tau_n$, $\lambda(\tau) = \lambda_n$ so that $p_\psi < 1$ if $\tau_n = 0$.

Polynomial infectiousness $\psi(\tau) = \lambda(\tau)\exp\big(-\int_0^\tau \lambda(\tau')d\tau'\big)$ where the infectiousness (hazard rate) is specified by a polynomial $\lambda(\tau) = c_0 + c_1\tau + c_2\tau^2 + \ldots$ with user-defined non-negative coefficients c_i.

Deterministic infection time Deterministic transmission time $\psi(\tau) = \delta(\tau - \tau_0)$ for constant τ_0.

3 The next reaction method

The next reaction method operates by maintaining a priority queue (Q) that always contains all future times at which an active link (that is, a link connected to an infected node) will attempt to transmit the disease. Each entry in the queue is represented by a tuple (t, s, i, j, w) where t is the time of the event, s the type, (T) transmission or (R) recovery (in the case of SIR or SIS), i the infecting node, j the node that is being infected and w the weight of link (i, j). The algorithm is initialized with a list of initial infection times $t_1, \ldots, t_m$ of certain nodes $n_1, \ldots, n_m$ (procedure EPIDEMICINIT). In addition to the priority queue Q, the algorithm tracks the times T_i of the latest infection, and the times R_i of the next recovery of node i; initially these times are set to $\perp$. Here, the symbol $\perp$ represents an undefined or uninitialized state.

```
procedure EpidemicInit((n_1, t_1), ..., (n_m, t_m))
    Q ← {(t_1, 'T', ⊥, n_1, ⊥), ..., (t_m, 'T', ⊥, n_m, ⊥)}
    T_i, R_i ← ⊥ for all nodes i
end procedure
```

At each step (procedure EPIDEMICSTEP), the algorithm retrieves the earliest event from the queue, processes it, and returns it. Transmission events cause the target node to become infected (procedure INFECTNODE) if transmission across the link is possible (procedure TRANSMITACROSSLINK) and the target node is susceptible. Transmission is always possible for static networks: the procedure TRANSMITACROSSLINK only blocks certain transmissions when simulating an epidemic on a temporal network, see Section 4. Successful transmission generate further events in the queue, which are then processed by later calls to EPIDEMICSTEP in order of their occurence. The procedure EPIDEMICSTEP is iterated until either the queue is empty (at which point the epidemic has stopped) or until some stopping condition is met.

```
procedure EpidemicStep(t_max)
    if Q is empty or has no entry with time t ≤ t_max then
        return (∞, ⊥, ⊥, ⊥, ⊥)
    end if
    fetch and remove event (t, s, i, j, w) with minimal t from Q
    if s = 'T' and TransmitAcrossLink(i, j) and node j is susceptible then
        InfectNode(t, j)
    else if s = 'R'and SIS then
        mark node j as susceptible
    else if s = 'R'and SIR then
        mark node j as recovered
    else
        start EpidemicStep from the top
    end if
    return event (t, s, i, j, w)
end procedure
```

Upon infection of a node i (procedure INFECTNODE), the infection time T_i is updated, a recovery time R_i is generated (for SIR and SIS models) and all of the node's outgoing links (i, j) are activated.

procedure InfectNode(t, i)
  mark node i as infected
  $T_i \leftarrow t$
  if SIR or SIS **then**
    $R_i \leftarrow T_i + \text{DrawTime}_\rho(0, 1)$
    add event $(R_i, \text{‘R’}, i, i, \perp)$ to Q
  end if
  for $l = 1, \ldots, \text{NodeDegree}(i)$ **do**
    $(j', w') \leftarrow \text{Neighbor}(i, l)$
    ActivateLink(t, i, j', w')
  end for
end procedure

Upon activation of a link (i, j) with weight w (procedure ActivateLink), an tentative infection time τ for node j is sampled and an infection event is added to the queue. For correctness on temporal networks, the time interval until infection is sampled from $\psi(\,\cdot\,|\, t - T_i, w)$ defined in section 2; this correctly handles links which are added retroactively after a node has already been infected. On static networks, $T_i = t$ and this condition is thus immaterial. If the infection time lies after node i's recovery time, no event is added since recovered nodes cannot spread the infection.

procedure ActivateLink(t, i, j, w)
  $\tau \leftarrow \text{DrawTime}_\psi(t - T_i, w)$
  if $t + \tau < R_i$ **then**
    add event $(t + \tau, \text{‘T’}, i, j, w)$ to Q
  end if
end procedure

The next reaction algorithm also permits to query the time of the next event without executing it, by inspecting the priority queue. This is not usually required for simulations on static networks, but it is crucial for simulations on temporal networks, see Section 4.

procedure EpidemicNext($t_{\max}$)
  if Q is empty or has no entry with time $t \le t_{\max}$ **then**
    return ∞
  else
    return time of earliest entry in Q
  end if
end procedure

3.1 Computational complexity

We consider an epidemic spreading on a network with basic reproduction number R_0, i.e. where an infected node on average causes R_0 subsequent infections. On such a network, we consider an epidemic with I infected nodes and thus at most $|Q| = IR_0$ active links. Assuming an appropriate data-structure such as a heap is used to represent the priority queue Q, the time complexity of adding an entry and removing the earliest entry from Q is $O(\log |Q|)$. Here, $|Q|$ denotes the number of queue entries, i.e. the number of active links. Under these assumptions the average time complexity of EpidemicStep is $\mathcal{O}(\log(IR_0))$.

4 Temporal networks

In the Main Text, we have defined temporal networks in terms of a function $\varepsilon_{ij}(t)$ which takes the value one if the network has a link from i to j at time j, and zero otherwise. Our algorithm adopts a computationally more efficient representation. Compared to static networks, we extend the abstract interface used to query temporal networks by two additional procedures:

NetworkStep($t_{\max}$) determines and executes the next change in network topology, i.e., moves to the next time at which one of the functions ε_{ij} jumps. Possible changes in topology are addition of a link, removal of a link, or an instantaneous contact between nodes. The time and type of change is returned in the form of a tuple (t, s, i, j, w) where t is the time of change, s the type ('+' for an added link, '−' for a removed link, '*' for an instantaneous contact), i and j are the source and target nodes, and w is the link's weight. Infinitesimal contacts correspond to δ-peaks of ε_{ij}; the probability of transmission during such a contact is thus $1 - \exp(-w\lambda(\tau))$. If the topology does not change by time $t_{\max}$, the procedure returns no event, i.e. $\perp$. After the procedure concludes, the topology as reported by NodeDegree and Neighbour reflects the reported change.

NetworkNext($t_{\max}$) returns the time of the next event without executing the event. Calls to this procedure thus leave the topology as reported by NodeDegree and Neighbour unchanged, and subsequent calls to NetworkNext report the same time until NetworkStep (or EpidemicStep if the network topology reacts to changes in epidemic state) is called. If no change in topology occurs until time $t_{\max}$, the procedure returns ∞.

Simulating epidemics on temporal networks

To simulate epidemics on temporal networks which may change in response to epidemic events, we rely on rejection sampling. Once a link has been activated, we do not reverse this activation before the link "fires", i.e., before its transmission time is reached. Instead, if a link adjacent to an infected node is removed, we mark the link as masked. When a link is masked, attempts at transmitting the disease through it are ignored. This avoids having to remove events other than the earliest one from the priority queue, which is an operation not typically supported by priority queues and likely costly.

The simulation algorithm otherwise reuses the simulation algorithm for static networks from Section 3 (or may indeed use any other simulation algorithm for which equivalent procedures EpidemicNext, EpidemicStep, InfectNode and ActivateLink can be provided).

```
procedure TemporalNext(t_max)
    return min(EpidemicNext(t_max), NetworkNext(t_max))
end procedure
```

The network is evolved in lock-step with the simulation of the epidemic. At any time, the time of the next event is thus the earlier of two times, the time of the next epidemic event (i.e. infection or recovery) and the time of the next network event (i.e. topology change), see procedure TemporalNext.

During each simulation step (procedure TemporalStep), the algorithm then performs either an epidemic step (similar to the static network case), or a network step (described above), depending in which time was earlier. The algorithm assigns a state to each link adjacent to an infected node: *admissible* (active and may transmit, or inactive and may be activated), *masked* (active, but transmissions are blocked), or *transmitted* (has successfully transmitted the disease). The general procedure goes as follows: When a new outgoing link is added to an already infected node, the link is activated. When an

```
procedure TemporalStep(t_max)
    if TemporalNext(t_max) = NetworkNext(t_max) ≠ ∞ then
        (t, s, i, j, w) ← NetworkStep(t_max)
        if node i is infected then
            if s = '+' and link (i, j) is admissible then
                ActivateLink(t, i, j, w)
            else if s = '+' and link (i, j) is masked then
                update link (i, j) to admissible
            else if s = '−' and link (i, j) is admissible then
                update link (i, j) to masked
            else if s = '*' and node j is susceptible then
                p ← 1 − exp(− w · HazardRate_ψ(t − T_i))
                InfectNode(t, j) with probability p
            end if
        end if
    else if TemporalNext(t_max) = EpidemicNext(t_max) ≠ ∞ then
        (t, s, i, j, w) ← EpidemicStep(t_max)
        if (t, s, i, j, w) = (∞, ⊥, ⊥, ⊥, ⊥) then
            start TemporalStep from the top
        end if
        initialize outgoing links (j, k) of infected/recovered node j to admissible
    end if
    return event (t, s, i, j, w)
end procedure
```

active link is removed, it is *masked*, which causes transmission attempts to be blocked. When a link is re-added while still *masked*, it reverts from *masked* to *admissible*. When a *masked* link attempts to transmit, it reverts back to *admissible* to indicate that it is now inactive and must be re-activated upon being re-added. When an *admissible* link transmits, it changes to state *transmitted* (see Fig. 2 in the Main Text).

The algorithm also implements instantaneous contacts. When an instantaneous contact from an infected node to a susceptible node appears, the susceptible node is infected with probability $1 - \exp(-w\lambda(\tau))$ where τ is the time since infection of the infecting node and w the weight of the contact. Such events are allowed only between nodes not currently connected by a link. As an optimization to reduce memory usage, link states are stored such that links in state *admissible* consume no memory.

The correct handling of *masked* links during transmission attempts is ensured by TransmitAcrossLink. This procedure blocks transmissions across *masked* links, and tracks whether links have already successfully transmitted the disease.

```
procedure TransmitAcrossLink(i, j)
    if link (i, j) is admissible then
        update link (i, j) to transmitted
        return true
    else if link (i, j) is masked then
        update link (i, j) to admissible
        return false
    end if
end procedure
```

The function TransmitAcrossLink is used by EpidemicNext, which was intro-

duced in Section 3.

4.1 Correctness

We now show that the NEXT-Net algorithm indeed generates transmission times with distribution $\psi_{i,j}(\tau \,|\, w\,;\, T_i) = w\lambda(\tau)\varepsilon_{ij}(T_i+\tau)\exp\left(-w\int_0^\tau \lambda(\tau')\varepsilon_{ij}(T_i+\tau')d\tau'\right)$ stated in Eq. (4) in the Main Text for link (i,j).

We fix a link (i,j) and first consider a modified version of the algorithm above, where ActivateLink is called whenever TransmitAcrossLink would update the state to *admissible* after a blocked transmission attempt, instead of delaying re-activation until the link reappears. We observe that after this modification, the successive invocations of ActivateLink incrementally generate tentative transmission times $\tau_1 \leq \tau_2 \leq \cdots$ (expressed relative to node i's infection time) by generating waiting times $\Delta_k \sim \psi(\cdot\,|w,\tau_{k-1}\,)$ between these attempts (i.e. $\tau_k - \tau_{k-1} = \Delta_k$, $\tau_0 = 0$). By definition of $\psi(\cdot\,|w,t\,)$, these times are the jumps of an inhomogeneous Poisson process with intensity $w\lambda(\tau)$ in order of occurrence. In particular, the number of jumps $J(I) = |\{\tau_k\}\cap I\}|$ within I thus follows a Poisson distribution with rate $\Lambda(I) = w\int_I \lambda(\tau)d\tau$ and $J(I_1)$, $J(I_2)$ are independent if I_1, I_2 are disjoint.

The (modified) algorithm rejects τ_k while $\varepsilon_{ij}(T_i+\tau_k) = 0$ and accepts the first τ_k where $\varepsilon_{ij}(T_i+\tau_k) = 1$. For notational convenience we introduce $T_\varepsilon = \{\tau\,|\,\varepsilon_{ij}(T_i+\tau) = 1\}$, and consider times $\{\tau_{k_m}\} = \{\tau_k\}\cap T_\varepsilon$ where $k_1, k_2, \ldots$ index the times τ_k with $\varepsilon_{ij}(T_i+\tau_k) = 1$ in order of occurrence. Let $J_\varepsilon(I) = |\{\tau_{k_m}\}\cap I\}|$ be the number of such times within I, then crucially $J_\varepsilon(I) = J(I\cap T_\varepsilon)$. It follows immediately that (i) if I_1, I_2 are disjoint so are $I_1\cap T_\varepsilon$, $I_2\cap T_\varepsilon$ and hence $J_\varepsilon(I_1)$, $J_\varepsilon(I_2)$ are independent, and (ii) $J_\varepsilon(I)$ follows a Poisson distribution whose rate $\Lambda(I\cap T_\varepsilon)$ by definition of Λ is $w\int_I \lambda(\tau)\varepsilon_{ij}(T_i+\tau)d\tau$. Therefore, $\tau_{k_1}, \tau_{k_2}, \ldots$ are the jump times of a inhomogeneous Poisson process with intensity $w\lambda(\tau)\varepsilon_{ij}(\tau)$, and τ_{k_1} is the first firing time of such a process. Consequently, τ_{k_1} is distributed according to $\psi_{i,j}(\tau\,|\,w\,;\,T_i)$. This proves the correctness of the modified algorithm.

We consider now the original algorithm where re-activation of link after a blocked transmission attempt is deferred until the link re-appears. The generated times $\tau_1 \leq \tau_2 \leq \cdots$ are not jump times of an inhomogeneous Poisson process with intensity $w\lambda(\tau)$ in this case. However, only jumps which would later be rejected because $\varepsilon_{ij}(\tau) = 0$ are omitted. Therefore, the properties of $J_\varepsilon(I)$ are the same for the original and the modified algorithm, and consequently the original algorithm generates τ_{k_1} with distribution $\psi_{i,j}(\tau\,|\,w\,;\,T_i)$ as claimed.

4.2 Computational complexity

On temporal networks, three terms contribute to the time complexity of TemporalStep. The first is the time required to maintain the link states; this is $\mathcal{O}(1)$ and typically negligible. The second is the time required by NetworkNext to determine when the next change in network topology occurs. The complexity of NetworkNext depends on the specific model of temporal network. For networks such as activity-driven networks or temporal Erdös-Renyi networks, either the Gillespie algorithm or a version of the next-reaction scheme can be used to simulate the network evolution; in this case the time complexity of NetworkNext is either constant (Gillespie) or logarithmic in the number of active elements (next reaction). For other types of networks such as Brownian proximity networks, however, the time can scale linearly with the number of nodes.

The third contribute to the complexity of TemporalStep are calls to EpidemicStep. A single call has time complexity $\mathcal{O}\big(\log(IR_0)\big)$ as discussed in Section 3.1. Each time TransmitAcrossLink encounters a *masked* link and blocks transmission, an additional epidemic step has to be performed. The total contribution of EpidemicStep

to TemporalStep is thus $\mathcal{O}\big(K \log(IR_0)\big)$ where K is the average number of transmission attempts required before encountering the link in state *admissible*.
